# Bone Mineral Density Evaluation Among Type 2 Diabetic Patients in Rural Haryana, India: An Analytical Cross-Sectional Study

**DOI:** 10.7759/cureus.45908

**Published:** 2023-09-25

**Authors:** Nitish Khandelwal, Surbhi Rajauria, Siddhesh Pandurang Kanjalkar, Omkar Shivaji Chavanke, Sanjay Rai

**Affiliations:** 1 Department of Pathology, Military Hospital Ambala Cantt, Ambala, IND; 2 Department of Pathology, Maharishi Markandeshwar University Mullana, Ambala, IND; 3 Department of Orthopedics, SMBT Medical Collage Igatpuri, Nashik, IND; 4 Department of Orthopedics, Military Hospital Ambala Cantt, Ambala, IND

**Keywords:** rural area, osteopenia, type 2 diabetes osteoporosis, qualitative ultrasound, bone mineral density

## Abstract

Background and objective

Diabetes is one of the most prevalent diseases globally, affecting almost all organ systems. The relationship between type 2 diabetes mellitus (T2DM) and bone mineral density (BMD) has been a matter of controversy, and data from developing countries in this regard is highly scarce. Early detection of low BMD in diabetic patients will help prevent further bone loss and risk of fragility fracture. In this study, we aimed to assess the effect of T2DM on BMD among the rural population of Haryana, India.

Materials and methods

This was a cross-sectional study involving 850 patients between 25 and 60 years of age, including 425 diabetic and 425 non-diabetic subjects (as controls). Calcaneus BMD was measured by using quantitative ultrasound (QUS), and the data were compared against matched parameters in both groups.

Results

The mean age of diabetics was 42.21 ± 10.5 years and that of non-diabetics was 42.18 ± 10.4 years. The mean BMI was 27.8 ± 4.17 kg/m^2^ in diabetics and 21.6 ± 3.32 kg/m^2^ in the non-diabetic control group. BMD values significantly differed between the groups: -4.3 ± 1.23 vs. -2.6 ± 0.34 in diabetics and non-diabetics, respectively (p=0.002).

Conclusion

A significant difference in BMD was observed between the diabetic and non-diabetic groups. Based on our findings, We recommend that all type 2 diabetics be screened for osteoporosis so that this silent bone loss can be detected in the early phase itself and appropriate preventive measures can be promptly initiated.

## Introduction

Diabetes and osteoporosis are common metabolic diseases. Diabetes has evolved into one of the most prominent healthcare issues globally and affected about 422 million people in 2014 [1]; many studies have reported that it affects several organs, including bone, which leads to a loss in bone mineral density (BMD) and the development of osteoporosis [2-6]. The latter is a silent metabolic disease with a detrimental effect on bone health, often resulting in fragility fractures. 

One of the most commonly utilized markers for the clinical diagnosis of osteoporosis is BMD. Bone quality is another significant biomarker of osteoporosis apart from bone density. Bone density is more frequently employed in clinical practice since poor bone quality is more difficult to detect. Osteoporosis can be detected even in cases where BMD fails to fulfill the diagnostic criteria provided there are fragility fractures in nearby structures. Hence, while BMD is not the "Gold Standard," it still remains one of the most commonly employed parameters for detecting osteoporosis. In this study, we hypothesized that low BMD would be observed in type 2 diabetes mellitus (T2DM) patients.

## Materials and methods

A cross-sectional analytical study was conducted involving 850 patients between 25 and 60 years of age, including 425 diabetic and 425 non-diabetic subjects. The study was commenced after obtaining Institutional Ethical Committee approval (letter no: MHA/Ortho/01/2023) and informed consent from the subjects. The study was conducted at a medical camp from July 2023 to August 2023. Patients with T2DM for more than five years were included in the study.

All patients and controls underwent detailed clinical examination and necessary blood investigations. Patients with chronic kidney disease, ischemic heart disease, primary hyperparathyroidism, thyroid function malabsorption syndromes, malignant diseases, autoimmune disease, inflammatory joint disease, and women with hysterectomy, as well as patients on steroids, any kind of hormone replacement therapy, immunosuppressants, or calcium and vitamin D supplements were excluded from the study. BMD was measured in the calcaneus using quantitative ultrasound (QUS), and the data were analyzed on the basis of the t-score and Z-score, using the WHO criteria [28]. Osteopenia and osteoporosis were considered to represent an abnormal BMD.

Quantitative ultrasound of the calcaneus

QUS was used because it is a non-invasive, portable, patient-friendly method for estimating the bone mass from the calcaneus bone. The mean QUS of the calcaneus was calculated for both feet. T-score and Z-scores were obtained by QUS, which was equal to the standard deviation (SD) of the mean value (stiffness) in non-diabetics of the same age and sex. The scores were considered as continuous variables during analyses. This test measures two parameters, firstly broadband ultrasound attenuation (BUA) and secondly, speed of sound (SOS), in addition to the T-score, which is comparable to that obtained by dual X-ray absorptiometry (DEXA).

We also calculated the stiffness index (SI) and estimated BMD (eBMD) parameters from both BUA and SOS. The SI and eBMD, expressed in percent and g/cm^2^, respectively, are variables obtained from a linear combination of BUA and SOS and calculated using the following equations. 

SI was calculated using the following formula, where 0.67 and 0.28 are constant coefficients, BUA is broadband ultrasound attenuation, SOS is the speed of sound, and 420 is the constant coefficient:

SI = (0.67 x BUA) + (0.28 x SOS) - 420

Similarly, eBMD expressed in g/cm^2 ^is derived from a linear combination of BUA and SOS and calculated according to the following formula, where 0.0025 and 3.68 are constant coefficients:

eBMD = (0.0025 x (BUA+SOS) - 3.68

Sample size calculation

We used the Cochran formula to calculate the required sample size. Assuming an acceptable margin of error of 5% and a 99% confidence interval, the sample size was determined to be 338 patients. By assuming a 10% dropout rate to minimize errors and increase reliability, a target sample size of 425 patients was determined.

Statistical analysis

We used Microsoft Excel 2019 and IBM SPSS Statistics version 19 (IBM Corp., Armonk, NY) for statistical calculation and analysis. BMD data of type 2 diabetic patients were compared with those of non-diabetic controls matched for age, using unpaired Student’s t-test.

Statistical analysis was performed using the Student’s t-test for paired data and unpaired parametrical data. Data were presented as mean ± SD or number unless otherwise specified. The Pearson correlation was used to evaluate the robustness of the BMD's relationship with other parameters. We used percentages and frequencies for categorical variables and, to find out th associations between categorical variables, a Chi-square (χ^2^) test was used.

## Results

The mean age of the diabetic patients was 42.21 ± 10.5 years while that of non-diabetics was 42.18 ± 10.4 years. The mean BMI was 27.8 ± 4.17 kg/m^2^ in diabetics and 21.6 ± 3.32 kg/m^2^ in the non-diabetic control group. No significant difference in terms of smoking, alcohol intake, serum alkaline phosphatase (ALP), serum calcium, and serum phosphorus levels between the groups was recorded. However, a significant difference was recorded between fracture episodes: 187 (44%) vs. 98 (23%) in diabetics and non-diabetics, respectively (p=0.002). A higher prevalence of hypertension was also recorded (59% vs. 21%) in diabetics compared to non-diabetics (p=0.003).

Also, higher serum creatinine levels were recorded in diabetics. A significant difference in BMD values was noted: -4.3 ± 1.23 vs. -2.6 ±0.34 in diabetics and non-diabetics, respectively (p=0.002). The mean hemoglobin A1c (HbA1c) value among diabetics was 8.0 ± 1.14% compared to 5.49 ± 0.55% in non-diabetics (Table 1).

**Table 1:** Demographic and clinical characteristics of the study population ^#^Categorical variables, expressed as frequency (percentage) of sample and p-values were derived from the Chi-square test; *p<0.05; ^@^continuous data are presented as mean ± standard deviation and p-values were derived from one-way analysis of variance (for continuous variables normally distributed); ^$^continuous data are presented as mean ± standard deviation and p-values were derived from Kruskal-Wallis test (for continuous variables not normally distributed) ALP: alkaline phosphatase; BMD: bone mineral density; BMI: body mass index; SD: standard deviation

Characteristics	Diabetic population	Controls	t	X^2^	P-value
Age, years, mean ± SD	42.21 ± 10.5	42.18 ± 10.4	1.15	-	0.786^@^
Sex, M:F	316:109	279:146	1.12	-	0.753^#^
HbA1c, %, mean ± SD	8.0 ± 1.14	5.49 ± 0.55	1.72	-	0.953^$^
BMI, kg/m^2^, mean ± SD	27.8 ± 4.17	21.6 ± 3.32	1.76	-	0.774
Serum calcium, mg/dl, mean ± SD	9.21 ± 0.23	9.01 ± 0.43	1.15	--	0.985
Serum vitamin D, ng/mL, mean ± SD	21.29 ± 46.17	36.32 ± 54.66	1.23	-	0.666
Serum ALP, mg/dl, mean ± SD	81.23 ± 11.43	86.33 ± 15.19	1.19	-	0.663^#^
Serum creatinine, mg/dl, mean ± SD	1.06 ± 0.87	0.4 ± 0.12	6.31	-	0.001^*^
Serum phosphorus, mg/dl, mean ± SD	2.5 ± 0.23	2.4 ± 0.68	1.12	-	0.887^#^
Alcohol consumption, %	49%	48%	-	1.76	0.961^#^
Smoking, %	29%	42%	-	0.56	0.871^#^
Hypertension, %	59%	21%	-	0.77	0.003^*^
BMD t-score, mean ± SD	-4.3 ± 1.23	-2.6 ± 0.34	4.56	9.42	0.051^*^
Z-score, mean ± SD	-0.99 ± 1.10	0.99 ± 1.09	3.12	-	0.897
Fragility fracture, n (%)	187 (44%)	98 (23%)	6.74	11.4	0.002^*^
Metformin	389	-	-	-	-
Pioglitazone	318	-	-	-	--
Combination	258	-	-	-	
History of fracture, %	12%	2%	-	-	0.241^#^

Of note, about 93% of diabetics and 56.6% of non-diabetics were noted to have low BMD. Among the entire study population (850), about 74.4% were noted to have low BMD. Among them, 62.2% were diabetics and 37.7% were non-diabetics. The remaining 25.2% had normal BMD: 13.9% were diabetics and 86.04% were non-diabetics. A significant difference was found between the groups with regard to BMD, as shown in Table 2.

**Table 2:** BMD distribution in both groups ^@^Low BMD includes both osteopenia and osteoporosis; *out of 425 patients; **out of the total population (n=850); ^#^Chi-square (χ2)=0.031, p=0.0231 BMD: bone mineral density

BMD	Group		Total population (n=850)	P-value
	Diabetic population	Non-diabetic population		
Normal, n (%)	30 (13.9%)	185 (86.04%)	215 (100%)	
	7%^*^	43.5%^*^	25.2%^**^	
Low^@^,n (%)	395 (62.2%)	240 (37.7%)	635 (100%)	0.0231^#^
	93.0%^*^	56.4%^*^	74.7%^**^	
Total	425	425	850	

The frequency of low BMD (osteopenia and osteoporosis) in calcaneus differed significantly between the diabetic and control groups (low BMD: 62.2% in diabetics vs. 37.7% in the non-diabetic control group, p=0.0231). The odds ratio (OR) for low lumbar spine bone density in normal people was 2.7 (CI: 0.8-8, p=0.1). There was a significant direct correlation between the bone density of calcaneus (r=0.64, p=0.00) in both groups.

On further analysis of abnormal BMD, osteopenia was found in 58.8% of diabetics and 41.1% of non-diabetics (p=0.023). Similarly, osteoporosis was observed in 66.6% of diabetics and 33% of non-diabetics (p=0.035) (Table 3).

**Table 3:** BMD distribution (normal, osteopenia, and osteoporosis) in both groups *Out of 425 patients; **out of the total population (n=850) BMD: bone mineral density

BMD	Group		Total population (n=850)	P-value
	Diabetic population	Non-diabetic population		
Normal	30 (13.9%)	185 (86.04%)	215 (100%)	0.012
	7.05%^*^	43.5%^*^	25.29%^**^	
Osteopenia	213 (58.8%)	149 (41.1%)	362 (100%)	0.023
	50.1%^*^	35.0%^*^	42.5%^**^	
Osteoporosis	182 (66.6%)	91 (33.3%)	273 (100%)	0.035
	42.8%^*^	21.4%^*^	32.1%^**^	
Total	425	425	850	

We performed a logistic regression analysis regarding the association between osteoporosis and non-obesity and obesity, the unadjusted OR for the overall association, and BMI with adjustment for age, HbA1c, sex, smoking, hypertension, and alcohol intake. The unadjusted OR for the principal association was 2.83 (95% CI: 1.12-6.84), indicating a statistically significant association between osteoporosis and BMI. Even after adjustment for age and smoking, the OR remained statistically non-significant. It was found that the association between type 2 diabetes and osteoporosis was statistically significant (OR: 0.623, p<0.001) (Table 4).

**Table 4:** The association between type 2 diabetes and osteoporosis based on BMI *Adjusted for HbA1c, age, sex, smoking, hypertension, and alcohol intake BMI: body mass index; CI: confidence interval; OR: odds ratio

Obesity present	Diabetes	Total	Osteoporosis		Unadjusted OR (95% CI)	P-value	Adjusted OR* (95% CI)	P-value
			Present	absent				
No	No	180	180	89	2.54 (1.06-6.71	0.034	2.83 (1.12-6.84	0.032
	Yes	167	258	17				
Yes	No	245	60	96	6.78 (1.82-21.14)	0.021	8.1 (1.90-27.33)	0.041
	Yes	258	137	13				

A gradual decrease in all the parameters of QUS such as BUA, SOS, eBMD, and SI was noted in both genders with increasing age. Higher values for all parameters were recorded in men as compared to women. Similarly higher values for all parameters, in both sexes, were detected in individuals aged <45 years.

Our results also showed significant negative correlations between T-score and QUS parameters in terms of age, sex, and menopausal age (p>0.05) (Table 5). On the other hand, we recorded a positive correlation between low T-score and QUS parameters in terms of higher HbA1c (p<0.05) (Table 5).

**Table 5:** Correlation of T-score and quantitative ultrasound scan values with sociodemographic data among diabetics (n=425) ^@^P-values were derived from one-way analysis of variance (for continuous variables normally distributed); ^$^p-values were derived from the Kruskal-Wallis test (for continuous variables not normally distributed); ^#^p-values were derived from the Chi-square test; *p<0.05. BMI: body mass index; BUA: broadband ultrasound attenuation; eBMD: estimated bone mineral density; SI: stiffness index; SOS: speed of sound

Variables	T-score	P-value	BUA	P-value	SOS	P-value	SI	P-value	eBMD	P-value
Age	-0.142	0.612	-0.173	0.632	-0.136	0.721	-0.176	0.833	-0.165	0.833^@^
Menopausal age	0.070	0.021^*^	0.147	0.021^*^	0.043	0.034^*^	0.114	0.015^*^	0.101	0.046^$*^
BMI	0.188	0.672	0.203	0.687	0.159	0.871	0.201	0.887	0.196	0.666^$^
HbA1c >7	0.187	0.013^*^	0.200	0.013^*^	0.159	0.001^*^	0.201	0.001^*^	0.196	0.001^#*^

## Discussion

Diabetes has been linked to changes in bone metabolism. It can result in aberrant bone metabolism and negative calcium metabolism, and can adversely influence sugar, protein, and fat metabolisms. While bone metabolism is influenced by T2DM, the association of T2DM with BMD remains inconsistent in the literature. Insulin-dependent diabetes mellitus does affect BMD. Many authors have noted varied effects. For instance, while Liu et al. [7] reported that type 2 diabetes increases BMD, Jang et al. [8] reported a decrease in BMD among diabetics. On the other hand, Sosa et al. [9] have reported that BMD does not alter with T2DM. As low BMD does not pose an immediate health problem, it usually goes unnoticed unless associated with fragility fractures. In a developing country such as India, many people are unaware of such an entity. Early detection of low BMD in diabetic patients may help prevent further bone loss and future risk of fracture.

In patients with long-standing diabetes, interpreting fracture data as a marker of bone health might be particularly challenging. Patients experiencing visual or neurological issues may be more vulnerable to accidents, which increases their risk of fracture, which is not always dependent on bone density alone. The existence of diabetic renal illness and autonomic and neuropathic alterations that could cause a loss of BMD and a low level of physical activity linked to diabetic problems are additional factors that make studies challenging to interpret [10,11]. Diabetes may affect the bone through several different methods, some of which may have contradictory effects. Obesity, which is common in people with T2DM, is highly correlated with greater BMD, most likely due to mechanical loading and hormonal factors such as insulin, estrogen, and leptin, as well as other hormonal factors [12,13,14]. Wakasugi et al. [15] have reported that a decrease in insulin secretion decreases BMD, and Yamagishi et al. [16] have reported that hyperinsulinemia increases BMD.

Singh et al. [17] and Vlassara and Uribarri [18] reported that in type 2 diabetics, advanced glycation end products (AGEs) are produced through various mechanisms. Yamagishi et al. [16] also noted that AGE-receptor for AGEs (AGE-RAGE) interactions serve as a causal link between diabetes and osteoporosis. Wongdee et al. [19] suggest that hypercalciuria in poorly controlled diabetes may result in renal hypercalciuria, which stimulates parathyroid, and thus the latter might contribute to osteopenia development in patients with T2DM. Furthermore, Park et al. [20] and Liu et al. [21] observed that bone remodeling and osteoblast differentiation may be negatively affected by non-enzymatic glycation of collagen. Subsequently, Yamamoto and Sugimoto [22] noted that buildup in the collagen fibers of bone, AGEs, a group of varied chemicals produced by a non-enzymatic interaction between reducing sugars and amine residues, physically alter the qualities of the bone substance, one of the causes of bone quality. Pritchard and Willett [23] have reported that high levels of pentosidine detrimentally affect bone strength.

Pittas et al. [24] in their meta-analysis noted that glycosuria in diabetics indirectly decreases BMD through the hypercalciuric effect, thereby creating hypocalcemia and further reducing BMD. Vezzoli et al. also noted similar results [25]. Fajardo [26] and Dhaon and Shah [27] reported that microangiopathy in type 2 diabetics reduces the blood flow in the bones, which may accelerate cortical thinning and fragility fracture by decreasing BMD.

The prevalence of osteoporosis, a common metabolic bone condition, among diabetic individuals adds to their illness burden. BMD is a marker for determining osteoporosis susceptibility [29,30,31,32,33]. A substantial difference in BMD between diabetic and non-diabetic individuals was seen in this study. Many researchers such as Dutta et al. [34], Xu and Wu [35], Yaturu et al. [36], and Zeid et al. [37] recorded low BMD in type 2 diabetics. However, Qiu et al. [38] and Ma et al. [39] noted that individuals with type 2 diabetes of both genders have higher BMD levels. Similarly, some other authors have also reported higher BMD in type 2 diabetics [40,41]. Furthermore, a few authors have reported no change in BMD in type 2 diabetes [9,42]. Recently, Naser et al. [43] found a high prevalence of low BMD (62.7%) and osteoporosis (18.8%) in T2DM patients, and they further noted a positive association between BMD and BMI, but a negative association between BMD and age. Later. Schwartz et al. observed that older women with T2DM have higher BMD but higher fragility fracture rates compared to non-diabetics [44].

Compared to controls, diabetic patients had lower serum ALP levels, which likely reflected decreased bone turnover [45,46,47,48] and increased osteoclastogenesis [49]. Marcucci et al. have noted that the increased oxidative stress in diabetic individuals has a negative impact on osteoblasts and may cause diabetic osteopenia [50]. It is well known that during active bone synthesis, ALP is produced by active osteoblasts. In diabetics, it is seen that dysfunctional osteoblastic activity leads to poor bone remodeling and overall low BMD, and, consequently, poor bone health [51,52]. In the present study, the majority of our patients had vitamin D deficiency, but we did not find any association between vitamin D levels and BMD.

In our multivariate analysis, calcium, phosphorus, and ALP did not correlate with BMD. Positive correlations between BMD at the spine and total cholesterol, LDL cholesterol, and triglycerides have been described previously. We did find a significant association between HbA1c (>7) and low BMD. On the contrary, Asokan et al. observed no association between HbA1c and BMD [6]. Many authors have noted that after a year of treatment, BMD decreased in the hip and spine, which may have been caused by the use of pioglitazone and insulin [53,54,55]. The use of pioglitazone is linked to the inability of mesenchymal stem cell precursors to commit to differentiating into osteoblast series [53,54].

In diabetes, oral anti-diabetic drugs seem to influence bone health and BMD in several ways. According to research, metformin inhibits cell division by upregulating cyclin D1 and activating AMP kinase, which in turn arrests the cell cycle [56]. Metformin use has also been linked to decreased levels of markers for bone growth [57]. Theoretically, this inhibitory effect on cellular proliferation could also affect bone progenitor cells, resulting in lower BMD with metformin, as shown in the current study. However, additional research is needed to fully understand this phenomenon.

In this study, the BMI did not differ between the two groups and did not predict BMD. The mean BMI of diabetic patients with normal BMD was 25.3 kg/m^2^, and that of patients with abnormal BMD was 24.1 kg/m^2^. Bilha et al. [58] concluded that women with long-standing type I diabetes have an increased risk for low bone mass. They also concluded that an increase in body weight partially affects BMD loss in T2DM. The present study recorded a negative association between diabetes duration, glycaemic control, and BMD. High serum creatinine was also observed in patients with low BMD.

Limitations of the study

The present study has a few limitations. Firstly, type 2 diabetic patients who were intermittently administered insulin to control blood sugar levels were not included. Secondly, the physical activity levels of the cohort were not recorded. Thirdly, no food practice or diet intake was recorded in this study. Finally, the parity status of the included women was not documented.

## Conclusions

In the present study, diabetic patients had lower BMD scores compared to non-diabetics, and the prevalence of osteoporosis was higher among diabetic patients. QUS is an effective and convenient tool to screen the diabetic population at large. Based on our experience and findings, we recommend that QUS be used for screening bone health in the local community, and it is advisable to validate its results against DEXA. For the most accurate detection of osteoporosis, QUS data should also be interpreted in conjunction with clinical risk factors. We found that diabetic patients had a higher risk of low BMD. Since osteoporosis is a preventable disease, the screening, early detection, and prevention of potential risk factors for osteoporosis in type 2 diabetics are crucial. It would be prudent to initiate an educational program for such individuals about healthy lifestyles that could potentially prevent or control osteoporosis, which includes raising awareness about physical activity, maintaining optimal weight, avoiding alcohol and smoking, eating a balanced diet, and regular sun exposure. This may contribute to healthy bones and improve bone density, thereby preventing fragility fractures among patients in developing countries.

Further studies with DEXA scans of the hip and lumbar spine are required to properly assess the t-score and to devise further management plans. Early detection of low BMD in these populations is critical in developing countries so that the risk of osteoporosis and subsequent fragility fractures can be minimized.
